# Toxicological studies of aqueous extract of Acacia nilotica root

**DOI:** 10.1515/intox-2015-0005

**Published:** 2015-03

**Authors:** Lukman Adewale Alli, Abdulfatai Ayoade Adesokan, Oluwakanyinsola Adeola Salawu, Musbau Adewunmi Akanji

**Affiliations:** 1 Department of Medical Biochemistry, College of Health Sciences, University of Abuja, Abuja, Nigeria; 2 Department of Biochemistry, Faculty of Basic Medical Sciences, College of Health Sciences, Ladoke Akintola University of Technology, Ogbomoso, Nigeria; 3 Department of Pharmacology and Toxicology, National Institute for Pharmaceutical Research and Development (NIPRD), Abuja, Nigeria; 4 Department of Biochemistry, Faculty of Science, University of Ilorin, Nigeria

**Keywords:** *Acacia nilotica*, acute toxicity, sub-acute toxicity

## Abstract

*Acacia nilotica* is a widely used plant in traditional medical practice in Northern Nigeria and many African countries. The aim of this study was to determine the toxicological effects of a single dose (acute) and of repeated doses (sub-acute) administration of aqueous extract of *A. nilotica* root in rodents, following our earlier study on antiplasmodial activity. In the acute toxicity test, three groups of Swiss albino mice were orally administered aqueous extract of *A. nilotica* (50, 300 and 2000 mg/kg body weight) and signs of toxicity were observed daily for 14 days. In the sub-acute toxicity study, four groups of 12 rats (6 male and 6 female) were used. Group 1 received 10 ml/kg b.w distilled water (control), while groups 2, 3 and 4 received 125, 250 and 500 mg/kg b.w of the extract, respectively, for 28 consecutive days by oral gavage. Signs of toxicity/mortality, food and water intake and body weight changes were observed. Biochemical parameters were analysed in both plasma and liver homogenate. In the acute and sub-acute toxicity studies, the extract did not cause mortality. A significant reduction in the activity of lactate dehydrogenase was observed at 250 and 500 mg/kg b.w, while alanine aminotransferase, aspartate aminotransferase and alkaline phosphatase activities were significantly higher than control values at 500 mg/kg b.w. The aqueous extract of *A. nilotica* was found to be safe in single dose administration in mice but repeated administration of doses higher than 250 mg/kg b.w of the extract for 28 days in rats may cause hepatotoxicity.

## Introduction

The use of plants in traditional medical practice for treatment of various ailments is usually regarded as harmless and safe in humans because they are derived from natural sources. This assumption is based on the common belief that herbs are by nature safer and gentler than drugs and plant-based medicine have been used in the treatment of diseases over many centuries (Newman & Cragg, 2007). An herb is just as prone to side effects as any medicine, especially when taken in high enough doses. Some medicinal plants may be safe at therapeutic doses, but those that are yet to be verified scientifically should be used with caution because they may cause adverse reactions when taken above recommended doses or when taken repeatedly over a period of time. Many studies have reported various toxic effects of herbal medicines, such as hepatotoxicity (Nwachukwu & Iweala, 2009) and nephrotoxicity (Colson & De Broe, 2005; Asif, 2012).

In Nigeria, the use of medicinal plants for treatment of different ailments is an essential part of traditional primary health care in many local communities. Yet prescription and use of some of these medicinal plants are not currently regulated, and thus there is a danger of inappropriate use, incorrect dosage and consequent adverse effects.

*Acacia nilotica (Linn.) Willd. Ex Del.* (*Fabaceae*) is an important plant used in traditional medical practice in Nigeria, many African countries and India (Bargal & Bargali, 2009). It is a scented, thorny, nitrogen fixing tree that grows to 14–17 m in height and 2–3 m in diameter. The leaves are small (2–5 mm long) and bipinnate consisting of 5–11 feather-like pairs. The pods are dark-green containing 8–12 ovoid seeds with a characteristic beaded necklace appearance (New, 1984). The root is usually brown in colour and of different sizes depending on the proximity to ground level. The leaves, fruits, bark and roots of *A. nilotica* are used locally in treatment of different diseases. African Zulu use the bark of *A. nilotica* to treat cough, diarrhoea, dysentery and leprosy (Van Wyk, 2000). The Massai (Kenya) use the bark and root decoction as aphrodisiac. The bark extract alone was reported (Agrawal *et al*., 2010) to increase the hepatocyte activity of antioxidant enzymes such as catalase, super-oxide dismutase, glutathione peroxidase and glutathione S-transferase. The aqueous extract of the bark was also documented (Eline *et al*., 2004) to increase milk production in lactating mothers. The fruit is used to treat tuberculosis (Oladosu *et al*., 2007), while the powdered pods are consumed by Egyptians to treat diabetes mellitus (Ali & Faruqi, 1969). In Northern Nigeria, the root is used for treatment of malaria (Etkin, 1997; Alli *et al*., 2011). This plant is a rich source of secondary metabolites such as alkaloids, terpenes, tannins, saponins and phenolics (Brenan, 1983, Alli *et al*., 2011). These secondary metabolites may be responsible for the various pharmacological activities of the plant extract in the treatment of diseases. The aim of this study is to investigate the acute and sub-acute toxicity of aqueous extract of *Acacia nilotica* root in Swiss albino mice and Wistar rats, respectively. The results obtained could be used to evaluate a possible human toxicity profile of repeated consumption of the aqueous extract of *A. nilotica* root for treatment of malaria and other ailments among the communities in the Northern Nigeria.

## Materials and methods

### Plant sample

Root sample of *A. nilotica* was collected around 8.45 a.m. at Chaza village, Suleja, Niger State, Nigeria. It was identified and authenticated by a taxonomist, Mrs Grace Ugbabe, at the herbarium of the National Institute for Pharmaceutical Research and Development (NIPRD), Abuja, where a voucher specimen number: NIPRD/H/6401 was deposited. The root material was air-dried at room temperature and pulverised into fine powder. The pulverised root was used to prepare fresh aqueous extract when needed.

### Preparation of aqueous extract

The aqueous extract of *A. nilotica* root sample was prepared by cold maceration as described by Adzu *et al*., (2003). Distilled water (2 L) was added to 500 g of powdered root sample and kept for 24 h with intermittent shaking, filtered first with muslin cloth and later with Whatmann filter paper. The filterate obtained from the extract was freeze-dried, using AMSCO/FINN-AQUA GT2 Freeze dryer (Germany). The dried extract (chocolate coloured crystals) was kept in a clean glass bottle and stored in the refrigerator at –4 °C until required for use. The yield was calculated with respect to the powdered root sample.

### Experimental animals

Swiss albino mice (*Mus musculus,* 25±2 g), and Wistar albino rats *(Rattus norvegicus,* 190±10 g) were used in this investigation. They were kept in well ventilated cages in the animal house facility of NIPRD under 12 h light/12 h dark cycle at a temperature of 25±2 °C. They were acclimatised for 7 days before onset of this study. Standard rodent pellet diet and water was provided *ad libitum*. All procedures used complied with the guidelines of the National Academy of Sciences (1996) on handling of experimental animals and ethical approval was obtained from the Animal Ethics Committee of NIPRD.

### Acute toxicity study

The acute toxicity of the aqueous extract of *A. nilotica* was evaluated in mice following the OECD Guidelines 423 (OECD, 2001). Four groups, with three female mice in a group, received the aqueous extract orally, at doses of 50, 300 and 2000 mg extract/kg body weight, respectively, while the control group received 10 ml/kg b.w of distilled water. The animals were observed individually after dosing for signs of toxicity (changes in skin, fur, respiration, motor activity) once during the first 30 min, periodically during the first 24 h and thereafter daily for 14 days. The LD_50_ value obtained from this study was used in estimating the various graded doses used in the sub-acute study.

### Repeated dose toxicity study

The twenty-eight-day sub-acute toxicity study was conducted in four groups of Wistar rats, consisting of six males and six females in each group, according to the OECD guideline 407 (OECD, 1995). Each of the three test groups received 125, 250 and 500 mg/kg b.w of the extract, respectively, while the control group received 10 ml/kg b.w of distilled water orally, for 28 consecutive days. All the animals were provided with standard rodent pellets and water ad libitum. They were observed daily for signs of toxicity and mortality. Cage side observations included changes in skin and fur colour, eyes, respiratory, motor activity and behavioural pattern. Attention was also paid to convulsion, tremor, salivation and sleep pattern. Water and food intake were measured daily by subtracting the left-over of water and food from the measured quantity provided the previous day. Body weight, average quantity of food and water intake was recorded every week.

### Clinical biochemistry

Blood, collected from the rats by cardiac puncture on the 29^th^ day in lithium heparin bottles, was centrifuged to obtain plasma which was used for clinical biochemistry assay. Liver homogenate was obtained after homogenisation of one gram of liver in 5 ml of 0.25 M ice cold sucrose solution (1: 5 w/v), as described by Akanji and Ngaha (1989). The homogenate obtained was centrifuged at 1000 × *g* for 15 min to obtain the supernatant, which was carefully transferred into clean sample bottles using a Pasteur pipette. Activity of some liver enzymes, *i.e.* aspartate aminotransferase (AST), alanine aminotrans-ferase (ALT), alkaline phosphatase (ALP) and lactate dehydrogenase (LDH), was determined in plasma and liver homogenates. Bilirubin, total protein, albumin, blood urea and creatinine, along with sodium and potassium ions, were analysed using biochemistry autoanalyser (Randox laboratories UK).

### Statistical analysis

Data were expressed as mean ± standard error of mean (SEM) of five replicates. Statistical analysis was done using Graphpad Prism version 4.00 (Graphpad software). The differences between the means were compared using analysis of variance (ANOVA) followed by Student’s t-test. *p*<0.05 was considered statistically significant.

## Results

### Acute toxicity study

The extract did not cause death or change in physical appearance and morphological characteristics in the treated animals throughout the 14-day observation period after single oral administration of 50, 300 and 2000 mg/kg doses of aqueous extract of *A. nilotica* in the acute toxicity study (Table 1). The estimated oral median lethal dose (LD_50_) in mice is 5000 mg/kg body weight.

**Table 1:** Toxicity and mortality during acute toxicity study of aqueous extract of *A. nilotica* in mice.

	Dose	No of mice with signs of toxicity/Normal behavior	No of mortality/Survival
Groups	(mg/kg b.w)	(ST/NB)	(D/S)
1 (Control)	0	0/3	0/3
2	50	0/3	0/3
3	300	0/3	0/3
4	2000	0/3	0/3

b.w = body weight, ST = sign of toxicity, NB = normal behaviour, D = dead, S = survive

### Body weight, food and water intake in repeated dose toxicity study

Oral administration of *A. nilotica* over 28 days produced an increase in absolute body weight during the experimental period, but there was no significant difference in body weight gain between the test groups and the respective control groups (Table 2). There was no significant reduction in food and water intake in either sex at all doses studied, compared with the control group (Tables 3 and 4).

**Table 2:** Effect of 28-day oral administration of *A. nilotica* on body weight of rats.

Treatments	Body weight of rats (g)			
(mg/kg b.w)	Week 1	Week 2	Week 3	Week 4
FEMALE				
Control	140.80±1.22	158.10±1.20	173.10±1.11	177.10±1.20
125	139.50±1.13	157.80±1.60	172.60±1.70	176.50±1.12
250	139.00±1.42	157.50±1.10	172.00±1.50	176.10±1.40
500	140.10±1.20	157.70±1.53	172.90±1.13	175.90±1.12
MALE				
Control	164.70±1.15	189.90±1.26	197.90±1.35	203.90±1.40
125	164.20±0.80	188.90±0.70	196.50±1.50	203.60±0.85
250	164.80±1.12	189.00±1.80	196.10±1.70	203.70±1.40
500	164.40±1.42	189.20±1.53	197.50±1.34	203.30±1.20

Values are expressed as Mean ± S.E.M of six observations

**Table 3:** Effect of 28-day oral administration of *A. nilotica* on food intake of rats.

Treatment	Food intake of rats (g/100g body weight)			
(mg/kg b.w)	Week 1	Week 2	Week 3	Week 4
FEMALE				
Control	10.40±1.25	9.00±0.63	9.10±0.87	9.30±0.47
125	10.30±1.76	8.80±0.78	9.00±0.47	8.90±0.72
250	10.10±1.34	8.70±0.85	9.00±0.39	8.80±0.59
500	10.10±1.09	8.60±0.74	8.80±0.46	8.80±0.59
MALE				
Control	10.40±1.39	9.20±1.67	9.40±0.79	9.50±0.68
125	10.40±1.46	9.00±1.29	9.10±0.85	9.20±0.71
250	10.20±1.13	8.80±1.17	9.40±0.81	9.10±0.46
500	10.20±1.14	8.70±0.89	9.20±0.83	8.90±0.75

Values are expressed as Mean ± S.E.M of six observations

**Table 4:** Effect of 28-day oral administration of *A. nilotica* on water intake of rats.

Treatment	Water intake of rats (ml/100g body weight)				
(mg/kg b.w)	Week 1	Week 2	Week 3	Week 4
FEMALE				
Control	16.80±1.70	16.40±1.24	16.30±0.85	16.40±0.56
125	17.10±1.65	16.50±1.47	16.40±0.88	16.10±0.57
250	17.20±1.84	16.50±1.51	16.30±0.75	16.20±0.88
500	17.20±1.92	16.20±1.36	16.00±0.69	16.00±0.85
MALE				
Control	18.00±1.92	17.50±0.88	17.20±0.68	17.40±1.42
125	17.90±1.65	17.30±0.75	16.90±0.58	16.80±1.15
250	17.70±1.84	17.60±0.85	16.90±0.58	16.80±1.15
500	17.70±1.75	17.20±0.79	16.80±0.58	16.90±1.63

Values are expressed as Mean ± S.E.M of six observations

### Clinical biochemistry

The effect of repeated oral dose of *A. nilotica* on the activity of some enzymes and on biochemical parameters of the treated animals is shown in Tables 5–8, 9 and 10, respectively. There was significant reduction in the activity of LDH at 250 and 500 mg/kg body weight, while the values obtained for ALT, AST, ALP and triacylglycerol were significantly higher than in controls at the dose of 500 mg/kg b.w. Concentration of cholesterol was significantly reduced at the dose of 500 mg/kg b.w compared to the control group.

**Table 5:** Effect of aqueous extract of *A. nilotica* on lactate dehydrogenase (LDH) activity in rats.

Treatment	LDH activity in Liver (IU/L)		LDH activity in Plasma (IU/L)(mg/kg b.w)	
(mg/kg b.w)	Male	Female	Male	Female
Control	798.00±2.64	795.60±3.54	80.50±1.76	79.70±1.54
125	784.90±2.05	782.70±1.04	78.00±1.84	77.00±1.62
250	661.20±1.22*	658.80±2.23*	66.90±1.28*	64.70±1.41*
500	654.80±2.56*	652.20±3.78*	60.80±1.15*	59.80±1.31*

Values are expressed as Mean ± S.E.M of six observations

* = significantly different from control at *p*<0.05

**Table 6:** Effect of aqueous extract of *A. nilotica* on alanine aminotranseferase (ALT) activity in rats.

Treatment	ALT activity in liver (IU/L)		ALT activity in plasma (IU/L)	
(mg/kg b.w)	Male	Female	Male	Female
Control	101.70±1.35	100.50±1.57	11.80±0.46	11.40±0.24
125	102.40±1.14	101.60±1.36	12.10±0.37	11.70±0.15
250	104.30±1.81	103.50±1.68	12.40±0.58	11.80±0.36
500	110.00±2.48*	108.00±1.26*	17.70±0.37*	17.20±0.59*

Values are expressed as Mean ± S.E.M of six observations

* = significantly different from control at *p*<0.05

**Table 7:** Effect of aqueous extract of *A. nilotica* on aspartate aminotranseferase (AST) activity in rats.

Treatment	AST activity in liver (IU/L)		AST activity in plasma (IU/L)	
(mg/kg b.w)	Male	Female	Male	Female
Control	274.80±1.35	268.50±1.57	17.00±0.46	16.60±0.34
125	270.50±1.14	270.30±1.36	17.50±0.37	17.30±0.25
250	280.60±2.81	273.00±2.68	19.00±0.58	18.60±0.46
500	303.30±2.48*	296.20±2.26*	19.80±0.37*	19.60±0.69*

Values are expressed as Mean ± S.E.M of six observations

* = significantly different from control at *p*<0.05

**Table 8:** Effect of aqueous extract of *A. nilotica* on alkaline phosphatase (ALP) activity in rats.

Treatment	ALP activity in liver (IU/L)		ALP activity in plasma (IU/L)	
(mg/kg b.w)	Male	Female	Male	Female
Control	168.70±1.67	168.10±1.45	27.00±0.69	26.60±0.47
125	170.20±1.65	170.80±1.43	27.30±0.18	27.10±0.31
250	180.00±1.59	179.50±1.37	28.00±0.47	27.60±0.69
500	181.90±2.65*	181.30±1.43*	28.50±0.55*	28.10±0.47*

Values are expressed as Mean ± S.E.M of six observations

* = significantly different from control at *p*<0.05

**Table 9:** Effect of aqueous extract of *A. nilotica* on blood biochemical values in repeated dose toxicity of male rats.

Parameters	Treatment			
	Control		Extract	
	10 ml/kg b.wt distilled water	125 mg/kg b.wt	250 mg/kg b.wt	500 mg/kg b.wt
Total protein (g/dL)	6.30±0.47	6.20±0.46	6.10±0.33	6.00±0.39
Albumin (g/dL)	3.80±0.49	3.70±0.48	3.60±0.24	3.50±0.27
Total bilirubin (pmol/L)	6.20±0.40	6.20±0.21	6.60±0.48	5.70±0.39
Conjugated bilirubin (pmol/L)	3.40±0.79	3.60±0.73	3.70±0.68	3.30±0.39
Cholesterol (mg/dl)	73.50±2.57	71.20±1.72	70.00±3.36	69.30±2.98*
Triacylglycerol (mg/dl)	53.20±1.42	54.20±0.64	55.80±0.65	57.80±0.58*
Sodium (mmol/L)	141.80±1.56	142.10±1.47	142.00±2.15	143.10±1.69
Potassium (mmol/L)	4.60±0.40	4.10±0.49	4.20±0.16	4.30±0.18
Urea (mmol/L)	8.70±0.54	8.40±0.45	8.60±0.43	8.90±0.45
Creatinine (mmol/L)	51.00±1.72	49.30±1.86	49.80±1.69	51.60±1.85

Values are expressed as Mean ± S.E.M of six observations

* = Significantly different from control at p < 0.05

**Table 10:** Effect of aqueous extract of *A. nilotica* on blood biochemical values in repeated dose toxicity of female rats.

Parameters	Treatment			
	Control		Extract	
	10 ml/kg b.wt distilled water	125 mg/kg b.wt	250 mg/kg b.wt	500 mg/kg b.wt
Total protein (g/dL)	6.10±0.25	6.00±0.24	5.90±0.26	5.80±0.17
Albumin (g/dL)	3.40±0.27	3.30±0.26	3.20±0.46	3.30±0.49
Total bilirubin (pmol/L)	5.70±0.28	6.00±0.19	5.30±0.26	5.50±0.17
Conjugated bilirubin (pmol/L)	2.50±0.57	2.70±0.63	2.90±0.46	2.60±0.48
Cholesterol (mg/dl)	71.50±2.35	69.00±1.94	67.50±2.76	66.20±3.14*
Triacylglycerol (mg/dl)	54.00±1.64	54.80±1.86	56.20±1.85	58.40±1.70*
Sodium (mmol/L)	141.00±1.78	141.50±1.69	142.00±2.07	142.70±1.47
Potassium (mmol/L)	4.20±0.38	3.90±0.27	4.00±0.38	4.10±0.39
Urea (mmol/L)	8.30±0.76	8.00±0.67	8.20±0.65	8.50±0.67
Creatinine (mmol/L)	50.40±1.50	48.50±1.64	49.20±1.47	50.40±2.07

Values are expressed as Mean ± S.E.M of six observations

* = Significantly different from control at *p*<0.05

## Discussion

Survival of mice after oral administration of 2000 mg/kg body weight of the extract (Table 1), up to fourteen days (observation period) implies that the estimated oral median lethal dose (LD_50_) of the extract at 5000 mg/kg body weight is non-toxic (OECD, 2001). According to the OECD 423 guideline, absence of mortality after oral administration of 2000 mg/kg b.w of the extract corresponds to a LD_50_ value of 5000 mg/kg. This suggests that acute oral administration of the extract is safe, and may also explain the reason why the root portion of the plant is widely used in traditional treatment of diseases.

Sub-acute toxicological evaluation of the aqueous root extract of *A. nilotica* was carried out in rats after the anti-plasmodial activity of aqueous root extract had been established in our previous study (Alli *et al*., 2011). It also enables us to assess the long-term toxicity profile of the extract and provides a guide for selecting a safe dose for further human use. There were no specific sex-related signs of toxicity and no mortality was recorded at any dose administered during the period of treatment. The survival of all the rats until the last day of treatment (28 days) confirms the safety of the aqueous root extract of *A. nilotica* in rats at the doses administered. There was no significant difference in the body weight of rats (Table 2), food intake (Table 3) and water intake (Table 4) at any of the doses administered, suggesting that the extract does not cause a significant change in appetite and consequently does not produce any significant difference in the weight of the treated animals when compared to the control. The change in body weight is a useful indicator of adverse effect of drugs/phytomedicines (OECD, 2001; Raza *et al*., 2002). Determination of food and water consumption is also an important component in the study of safety of any therapeutic agent. Proper intake of nutrients is essential for maintenance of the physiological status of the animal receiving a drug and also for a proper response to the drug tested, while inadequate nutritional intake may yield a wrong response (Ramesh *et al*., 2007). Analysis of liver function parameters in rats is a relevant part of toxicity evaluation because changes in these parameters could be a valuable indicator of possible organ toxicity (Zimmerman & Ishak, 1979; Olson *et al*., 2000). Determination of the activities of various enzymes in tissue and body fluid is also a significant part of toxicological investigation and a pointer to possible tissue damage (Akanji & Ngaha, 1989). The assay for some marker enzymes in this study was based on the specific location of these enzymes in the cell, since the site of cellular injury could be determined by evaluating the activities of these marker enzymes (Adesokan & Akanji, 2003). These enzymes were assayed in liver homogenate and plasma, and the changes in enzyme activity in the liver were compared to those in plasma.

Lactate dehydrogenase (LDH) is an intracellular (cyto-plasmic) enzyme that catalyzes the reversible conversion of pyruvate to lactate with concomitant generation of NADH from NAD^+^ in the anaerobic glycolytic pathway (Delvin, 2006). The significant reduction in LDH activity in the liver homogenate and plasma at 250 and 500 mg/kg body weight (Table 5) may be due to enzyme inactivation or reduced synthesis in the liver, resulting in reduced secretion into plasma. Considering the fact that LDH is an essential enzyme found in the energy generating anaerobic glycolytic pathway of many parasitic organisms, the inhibition of this enzyme could be a possible mechanism of anti-plasmodial and pharmacologic activities of the aqueous extract of *A. nilotica* root.

The aminotransferases (ALT & AST) are predominantly cytosolic liver enzymes (though some AST isoform can be found in the mitochondria) involved in transamination reactions during amino acid metabolism (Delvin, 2006). The significant elevation of ALT (Table 6) and AST (Table 7) in liver homogenate and plasma following administration of 500 mg/kg aqueous extract of *A. nilotica* may be due to enzyme induction by the high dose of the extract, resulting in increased synthesis of these enzymes (Zimmerman & Ishak, 1979) or it may be due to inflammatory injury to the cell membrane with consequent leakage of these cytosolic enzymes (Adesokan & Akanji, 2003). The elevated level of these enzymes may increase the rate of transamination reaction and subsequent catabolism of amino acids in the liver (Pagana & Pagana, 1998).

Alkaline phosphatase (ALP) is a marker enzyme for the plasma membrane and endoplasmic reticulum (Akanji *et al*., 1993). Alteration in the activity of this enzyme in tissues and plasma can be used to assess the integrity of the plasma membrane and it may suggest damage to the plasma membrane (Akanji & Yakubu, 2000).

The significant, dose dependent increase in ALP activity in the liver homogenate and plasma (Table 8) following administration of 500 mg/kg body weight extract, may be due to increased functional activity of the liver leading to increased synthesis of the enzyme or to membrane labilization, with consequent leakage of the enzyme into the plasma. Since ALP hydrolyses phosphate monoesters, the increase in enzyme activity could present a threat to cells that are dependent on phosphate esters for their vital metabolic processes and it may cause hydrolysis of phosphate ester metabolite of the liver (Akanji & Yakubu, 2000).

The total protein and albumin concentrations in plasma were not significantly altered when compared with the control in any of the doses administered (Tables 9 and 10). The fact that concentration of total protein and albumin were not altered, when compared with the control, suggests that the different doses of the extract used do neither produce any adverse effect on the synthetic functions of the liver nor do they cause kidney damage that would result in leakage of these proteins into the urine (proteinuria). Nor did the extract produce a significant difference in bilirubin concentration in the treated rats, as compared with the control.

Determination of serum electrolytes, urea and cre-atinine is an index of renal excretory function and can be used to diagnose impaired renal function (Crook, 2006). The absence of a significant difference in the concentration of any of the renal function parameters analyzed at any of the doses of the extract administered, compared with the control, implied that the extract does not have adverse effects on renal function at the doses tested (Tables 9 and 10).

## Conclusions

The aqueous extract of *A. nilotica* did not cause any adverse effects in single dose administration. Neither did the 28-day administration of repeated doses of 125 and 250 mg/kg b.w of the extract produce significant toxicological changes in the parameters under this study, except the reduced activity in LDH observed at 250 mg/kg b.w. administration. However, intake of the higher dose of 500 mg/kg b.w of the extract may have a hepatotoxic effect, based on the significant increase in the activity of AST, ALT and ALP. Therefore these enzymes should be monitored in cases using the extract for 28 days or more. No evidence of nephrotoxicity was observed from repeated administration of *A. nilotica* extract. Based on these results, it can be concluded that the aqueous extract of *A. nilotica* is not toxic after single dose administration and the no observable adverse effect level (NOAEL) is <250 mg/kg b.w. after 28-day repeated administration.
